# Novel monoclonal antibody-based immunochromatographic strip for detecting citrinin in fruit from Zhejiang province, China

**DOI:** 10.1371/journal.pone.0197179

**Published:** 2018-05-09

**Authors:** Haiwei Cheng, Yi Yang, Yifei Chen, Xueqiu Chen, Zizheng Cai, Aifang Du

**Affiliations:** 1 Institute of Preventive Veterinary Medicine & Zhejiang Provincial Key Laboratory of Preventive Veterinary Medicine, College of Animal Sciences, Zhejiang University, Hangzhou, China; 2 National Research Center of Engineering and Technology for Veterinary Biologicals, Ministry of Agriculture, Key Laboratory of Veterinary Biological Engineering and Technology, Jiangsu Academy of Agricultural Sciences, Nanjing, China; 3 Jiangsu Co-innovation Center for Prevention and Control of Important Animal Infectious Diseases and Zoonoses, Yangzhou, China; 4 Nanjing Agricultural University, Nanjing, China; Consiglio Nazionale delle Ricerche, ITALY

## Abstract

Citrinin (CIT) is a hepato-nephrotoxic fungal metabolite produced by the genera *Penicillium*, *Aspergillus* and *Monascu*. There is an increasing demand for rapid and economical methods for detection CIT residues in fruit. In this study, we developed an immunochromatographic strip (ICS) for detection of citrinin (CIT) residues in fruit for the first time. Anti-CIT monoclonal antibody (McAb) 2B9 was prepared, with a binding affinity of 9.39 × 10^8^ L/moL. Conjugates CIT-BSA and McAb 2B9 were used to develop the ICS which could be completed in 5 min, with the detection limit of 50 ng/mL and no cross reactivity with other mycotoxins. Analysis of CIT in 64 fruit samples revealed that data obtained from the ICS test were in good agreement with indirect competitive enzyme-linked immunosorbent assays (ic-ELISAs) and high performance liquid chromatography (HPLC). This result demonstrated that the ICS test could be used as a rapid, reliable, cost-effective and user-friendly qualitative tool for detection of CIT residues on-site.

## Introduction

With the improvement of living standards, food safety administration and research towards agricultural products in recent years are primarily focused on the pollution residues detection of heavy metal [1], microbial contaminants [2], antibiotics [3], pesticide [4, 5] and toxins [6, 7]. Mycotoxins are toxic metabolites generated by fungi under favorable environmental conditions. About 25% food crops are affected by mycotoxin contamination every year, causing considerable financial loss [8]. In addition, the presence of mycotoxins in animal feed is one of the most common problems in animal husbandry for their toxic effects in animals. Citrinin (CIT) (S1 Fig) is a hepato-nephrotoxic fungal metabolite produced by the *Monascus* [9], *Aspergillus* [10] and *Penicillium* [11], first isolated in 1931. CIT was used as an experimental antibiotic in the clinic [12]. However, researches indicated CIT often occurs in naturally contaminated agricultural products such as corn, wheat, apple and other agricultural products [13–16]. As a nephrotoxin in animals, CIT damages the proximal tubules of the kidney and was implicated as a potential causative agent for Balkan nephropathy [17]. It is well known to be one of the main mycotoxins that could result in several serious health problems. Consequently, most countries have set a legal limit for CIT, the allowed level of citrinin in red fermented rice in Japan is 200 μg/kg [18] and 100 μg/kg in the European Union [18]. China has set legal limit of 50 μg/L for CIT in liquid samples, and 1 mg/kg in solid samples [19].

Classical analytical techniques for detection of CIT residues include high-performance liquid chromatography [20], liquid chromatography with mass spectrometry [21], gas chromatography with mass spectrometry [22] and enzyme-linked immunosorbent assay [23]. However, chromatographic methods are unsuitable for processing of large quantity of samples on-site as they require trained personnel, expensive instruments, well-equipped laboratories, tedious sample preparation and long operation times. Therefore, there is an increasing demand for rapid and economical methods for determining CIT residues.

Colloidal gold has been widely used during the immunoassays and the colloidal gold particles, the assay can induce a visible color reaction once the colloidal gold particles-labeled antibody responds to the corresponding antigen [24]. Compared with other methods, the colloidal gold immunoassay could be obtained within 10 min with the advantages of no instruments requirements, easy operation and simple judgment [25]. Nowadays, the ICS test has been widely accepted as a useful tool for the detection of some mycotoxins, such as ochratoxin A [26], fumonisin B_1_ [27], aflatoxin B_1_ [28], deoxynivalenol [29, 30] and zearalenone [6]. The ic-ELISA method and ICS test for citrinin in cereals and red yeast rice have been reported [31, 32], however, previous researchers did not mention their detection in fruit samples and the detection time during the studies. Many other researches also indicated CIT often occurs in naturally contaminated fruit samples such as orange, apple and others [14–16].

In this study, we developed the ICS for detection of CIT residues in fruit for the first time. A highly specific anti-CIT McAb 2B9 was generated and a colloidal gold immunoassay was developed based on McAb 2B9 to detect CIT residues in fruit samples. Both spiked and natural samples were investigated by the ICS test which could be completed in 5 min, with the detection limit of 50 ng/mL and no cross reactivity with other mycotoxins. The ICS test results showed strong agreements with those obtained from the HPLC and ELISA analysis. We demonstrated in this study that CIT could be detected rapidly by ICS test in fruit samples collected from several regions of Zhejiang Province during 2016 and the ICS test could be used by personnel to screen samples fast and selectively without any training.

## Materials and methods

### Reagents and chemicals

Citrinin (CIT), Aflatoxin B1 (AFB1), Fumonisin B1 (FB1), Patulin (PAT), Ochratoxin A (OTA), polyethylene glycol (PEG) 1450, hypoxanthine aminopterin and thymidine (HAT) medium, hypoxanthine-thymidine (HT) medium, complete and incomplete Freund adjuvant were purchased from Sigma (USA). The mouse McAb isotyping kit was purchased from Hycult Biotechnology (Netherlands). The SP2/0 myeloma cell line was stored in our lab. The Ridascreen Fast CIT ELISA kit was purchased from the R-Biopharm group (Germany). Goat anti-mouse IgG (H+L) was purchased from BioWorld (USA). Protein-G Sepharose Fast Flow Columns were purchased from GE Healthcare (USA). Chloroauricacid (HAuCl_4_) and trisodium citrate were obtained from Shanghai Chemical Reagents (Shanghai, China). The nitrocellulose membranes (Millipore 135, Millipore 180) were purchased from Millipore (USA). Othertypes of nitrocellulose membranes (Sartorius CN 140, PALL Vivid 170), conjugation pads (GL0194, Ahlstrom 8964, Ahlstrom 6613), sample pads (GL-b01, GL-b02) and absorbent pads were purchased from Jieyi Bio. Co. (China).

### Apparatus

A Shimadzu technologies LC-20AT series system contained FLD detector (Japan) was used to perform the HPLC analysis. The transmission electron microscope system was JEM 1200EX from JEOL (Japan). The XYZ Biostrip Dispenser and CM 4000 Cutter were purchased from Bio-Dot (USA). The Phenomenex (USA) Gemini 5μm C_18_ reversed-phase column (4.6 mm × 150 mm, 5 μm) was used as the analytical column.

### McAb generation and characterization

The McAbs against CIT were prepared and characterized [23, 33–37]. Ascites fluids were produced and purified according to the instructions of the manufacturer. The details were provided in S1 File.

Mice used in this study were treated in strict accordance with the recommendations in the Guide for the regulation for the Administration of Affairs concerning Experimental Animal of the People’s Republic of China. Animal experiments were approved by Zhejiang University Experimental Animal Ethics Committee (Permit Number: ZJU201308-1-10-072). Mice were treated with plenty of water and food, living in a suitable habitat, without psychological trauma and extra pain during the course of our study. Mice were humanely euthanized under sodium pentobarbital anesthesia, with all efforts made to minimize suffering, and no mice showed adverse clinical signs during the course of our study.

### Preparation of colloidal gold

Colloidal gold with an average diameter of 25 nm was prepared according to the methods described by Bassab and Syamal [38] with minor modifications. Initially, 100 mL 0.01% tetrachloroauric acid was heated to boiling with a uniformly stir and 0.7 mL of 1% sodium citrate was quickly added. The mixture was kept stir uniformly for 20 min to stabilize the colloidal gold nanoparticles. The diameter and the dispersity of the nanoparticles were checked by transmission electron microscope (TEM) and the colloidal gold suspensions were stored at 4 ^o^C with 0.05% (w/v) sodium azide until use.

### Preparation of colloidal gold-McAb conjugates

0.5 mg/mL purified McAb 2B9 was added to different pHs of colloidal gold solution (pH 6.0, 6.5, 7.0. 7.5, 8.0, 8.5 and 9.0) to identify the most appropriate pH value for binding with the colloidal gold nanoparticles, respectively. As different pH values would induce different color reaction, the pH value of the sample showing no color changes was the most appropriate pH value for binding.

After the confirmation of the optimum pH value, different concentrations of McAb 2B9 (2, 4, 6, 8, 10, 12 and 14 μg) were added to the colloidal gold solution to determine the optimal concentration for binding The lowest concentration of McAb 2B9 of the sample showing no color changes was the most appropriate concentration for binding [39].

The colloidal gold nanoparticle labeled antibody was prepared according to the methods described by Roth [40] with slight modifications. A volume of 500 μL 0.5 mg/mL McAb 2B9 was added to 20 mL colloidal gold solution drop by drop followed by stirring gently for 30 min. 2 mL 10% (w/v) BSA was added and reacted for 15 min to stabilize the colloidal gold nanoparticles. Then the mixture was centrifuged at 1500 rpm at 4 ^o^C for 30 min and the pellet was dissolved in 20 mL 2 mM borate buffer (pH 7.5, with 1% BSA). Repeated the centrifugation and dissolution for three times to obtain pure colloidal gold nanoparticle labeled antibody, which was resuspended in 2 mL conjugate storage buffer (2 mM pH 7.5 borate buffer, including 4% sucrose, 6% trehalose, 1% BSA and 0.05% sodium azide) and stored at 4 ^o^C until use.

### Preparation of immunochromatographic strip

The preparation of immunochromatographic strip (ICS) was performed according to the method described by Xu et al. [29] with modifications. The aim of the ICS is to visualize the detection, thus the color intensity of the test line should be strong enough to observe and distinguish the positive samples from the negative ones. To achieve the most appropriate performance, all the components of an ICS would be evaluated individually. Different sources of nitrocellulose membranes (Millipore 135, Millipore 180, PALL Vivid 170 and Sartorius CN 140), conjugation pads (Ahlstrom 6613, Ahlstrom 8964 and GL0194) and sample pads (GL-b01 and GL-b02) were tested.

Different concentrations of conjugates CIT-BSA and CIT-OVA dissolved in 50 mM PBS (pH 7.5) including 10% methanol were applied, respectively. The conjugates and goat anti-mouse IgG antibody (1 mg/mL) were sprayed on the NC membrane at 2–3 μL/cm to be the test line (T line) and control line (C line), respectively. The distance between the two lines was 4–5 mm. Different sample pads and conjugate pads were pretreated with 50 mM pH 7.5 borate buffer (containing 5% trehalose and 1% BSA) and vacuum dried for 4 h. Then 10 μL colloidal gold-labeled anti-CIT McAb was added to the conjugate pad and air dried for 3 h.

The components of the ICS were assembled as described by Zhu et al. [41] with minor modifications, NC membrane was pasted on the backing card and the conjugate pad was overlaid with 2–3 mm-overlap with the NC membrane. Followed by the sample pad, adhered with 2–3 mm-overlap on the conjugate pad and finally the absorbent pad was put on the top. The whole assembled strips were cut longitudinally into strips (4 mm × 60 mm) and stored under dry conditions until use.

### Specificity and sensitivity of ICS

Samples containing 200 ng/mL of different toxins, such as Patulin (PAT), Aflatoxin B1 (AFB1), Fumonisin B1 (FB1) and Ochratoxin A (OTA) were also assayed to evaluate the specificity of the ICS. For the sensitivity assay, different concentrations of CIT (from 0 to 50 ng/mL) diluted by 50 mM pH 7.5 PBS (including 10% methanol and 0.05% Tween-20) were assayed to determine the detection limit. All the detections could be completed within 5 min and results could be inspected visually.

### Samples analysis

HPLC assay was performed first according to the method described in the National Standard to identify whether there was CIT residue in fruit samples. Different concentrations of CIT (30, 60, 120 ng/mL) were added to the negative samples to prepare the spiked samples. In total, 64 fruit samples were collected from different regions of Zhejiang province in 2016. For the icELISA and HPLC test, 10 g of the sample was dissolved in 100 mL of 70% ethanol solution and treated with an ultra-sonicator at 300 W for 15 min, then centrifugation at 3000 rpm at 4 ^o^C for 20 min. The supernatant was evaporated and the pellet was redissolved with 1 mL of 70% ethanol solution. The sample was then diluted with 10% methanol solution and detected by the Ridascreen Fast CIT ELISA kit. The ethanol extract was analyzed by HPLC with a volume of 50 μL. For the colloidal gold immunochromatographic strip test, 10 g of the sample was dissolved in 100 mL of 70% ethanol solution and shaked violently at room temperature for 30 min. The supernatant was filtered using the Whatman 2V filter and diluted 3 times by 50 mM PBS (10% methanol,0.05% Tween-20). Finally, 200 μL solutions were used for detection and the results were determined in 5 min. All samples were tested by the ICS and confirmed by the commercial ELISA kit and HPLC analysis.

## Results and discussion

### McAb preparation and characterization

To induce immunoreactions, CIT was conjugated to prepare the immunogen (CIT-BSA) and coating antigen (CIT-OVA), respectively. 6 mice were injected with CIT-BSA for 6 times and the serum titers were determined by indirect ELISA (S2 Fig). Mouse 1 had the highest titer and was chosen for cell fusion. Splenocytes isolated from mouse 1 were fused with SP2/0 myeloma cells by PEG 1450 and indirect ELISA was used to screen hybridoma cells against CIT. Four hybridoma cells named 3B11, 2B9, 2C1 and 4D5 were obtained (S3 Fig) and isotyping showed 3B11 and 2B9 belonged to the IgG1 subclass, 2C1 and 4D5 belonged to the IgG2a subclass (S4 Fig).

The sensitivity, specificity and affinity constants of the McAbs were characterized to choose the most appropriate McAb for the development of ICS. The sensitivity and specificity of the McAbs were measured by indirect competitive ELISA and the binding of McAbs to CIT-OVA was inhibited by different concentrations of free CIT, which indicated the four McAbs were specific to CIT (S5 Fig). McAb 2B9 showed the best IC_50_ of 85.29 ng/mL, while the IC_50_ of 3B11, 2C1 and 4D5 was 150.83 ng/mL, 113.46 ng/mL and 159.61 ng/mL, respectively. The limit of detection (LOD) was 7.98 ng/mL for 2B9, while the LOD of 3B11, 2C1 and 4D5 was 14.92 ng/mL, 15.79 ng/mL and 20.78 ng/mL, respectively. After the tests, McAb 2B9 showed the highest sensitivity. The cross-reactivity (CR) value was measured to evaluate the specificity, McAb 2B9 failed to react with other mycotoxins except CIT and all the CR values were less than 0.01% (S1 Table), indicating that McAb 2B9 was highly specific to CIT. The affinity constant *K*_*aff*_ of the McAb 2B9 was determined by indirect ELISA, with both the antigen CIT-OVA and the McAb 2B9 were serially diluted. After calculation, McAb 2B9 has the affinity of 9.39 × 10^8^ L/moL (S6 Fig). McAb 2B9 was chosen for the development of ICS for its high sensitivity, specificity and affinity.

The average chromosome numbers of splenocytes and SP2/0 myeloma cells were 36–40 and 61–70, respectively, and there were 102±4 chromosome in one hybridoma cell (S7 Fig). In order to avoid affecting the sensitivity of the ICS assay, McAb 2B9 must be purified before conjugation with colloidal gold particles [29]. In this study, McAb 2B9 from the ascites fluid was purified by a Protein-G Sepharose Fast Flow Column. SDS-PAGE analysis showed that the purification process was effective, yielding clear heavy chain and light chain with the molecular weight of 45 kDa and 25 kDa, respectively (S8 Fig). The titer of purified ascites was 1: 1,638,400 as determined by indirect ELISA (S9 Fig).

### Identification of colloidal gold

**Fig 1:**
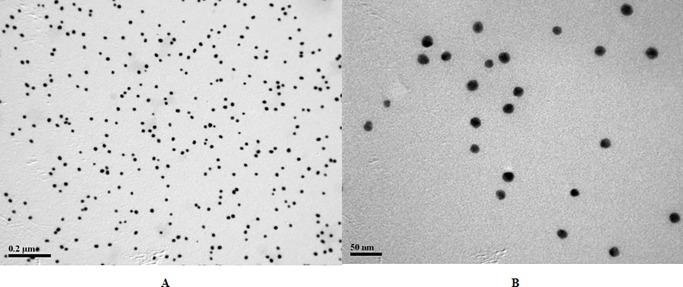
The size distribution of unconjugated colloidal gold nanoparticles by TEM. For particle size comparison, the 0.2 μm and 50 nm-scale markers are indicated in A and B, respectively.

The 25 nm colloidal gold nanoparticles were the optimal particle size for most diagnostic applications [27, 42] and were prepared in this study. The colloidal gold nanoparticles in solutions were homogeneous as observed by TEM, with their diameters between 20 nm and 30 nm (Fig 1). The immunoprobes remained stable and displayed clear color on the NC membrane after combining with an optimal amount of McAb. The results indicated that the colloidal gold was effective and could be used for the development of the ICS.

### Development and optimization of strip

The ICS was developed to be a qualitative assay with rapid visual evaluation to detect contamination at a threshold level. Thus the color intensity of the test line and control line must be high enough to be seen and enable observation of the difference between negative control and test samples. To develop the ICS, different parameters including the pH of colloidal gold solution, the amount of McAb, the CIT-protein conjugates and NC membrane were optimized according to the method described by Xu et al. [29].

As determined by the salt-induced precipitation method in this study, the most appropriate pH for labeling was found to be 8.5 and the optimal amount of McAb 2B9 for labeling was 12 μg/mL in colloidal gold solutions with the pH of 8.5.

Two CIT-protein conjugates (CIT-BSA and CIT-OVA) were used to increase strip sensitivity, and the CIT-BSA conjugates were identified to yield higher signals and better sensitivity than CIT-OVA, with the optimum concentration of 0.15 mg/mL. The reasons could be referred to previous literatures [27, 29]: 1, BSA having a higher coupling ratio than OVA in the coupling reaction with CIT; 2, BSA having higher stability while coupling with CIT; 3, CIT-BSA could improve assay sensitivity as the lower concentrations of coating antigen used, the better cut-off limit of antigen generated.

As different materials could affect the sensitivity, signal brightness, test time and other parameters of the ICS, the selection of NC membrane, conjugate pad, sample pad and extraction solution were established by color intensity of the T line and C line on different materials. It was demonstrated that M180 produced highest color intensity of T line as compared with others and gave best sensitivity in 5 min without flow disturbance, which suggested that during this study M180 may be more effective during the binding of the protein conjugate and the slower nominal flow rates of M180 may affect the interaction of the colloidal gold-labelled anti-CIT McAb with the CIT-BSA on NC membrane. Ahlstrom 8964 was the appropriate conjugate pad for its performance in releasing and protecting the colloidal gold labeled antibody. Ahlstrom 8964 was pretreated with trehalose and BSA in this study, because it could reduce the non-specific background signal in ICS test [27, 29, 30]. GL-b01 was chosen as the suitable sample pad due its ability to hold more determining samples compared to GL-b02, and GL-b01 can increase the color of the test strip. In addition, high concentration of methanol in the extraction solution could damage the NC membrane and affect the immunoreactions, so 10% (v/v) methanol-PBS buffer was chosen in our assay [23].

### Principle of immunochromatographic strip test

**Fig 2:**
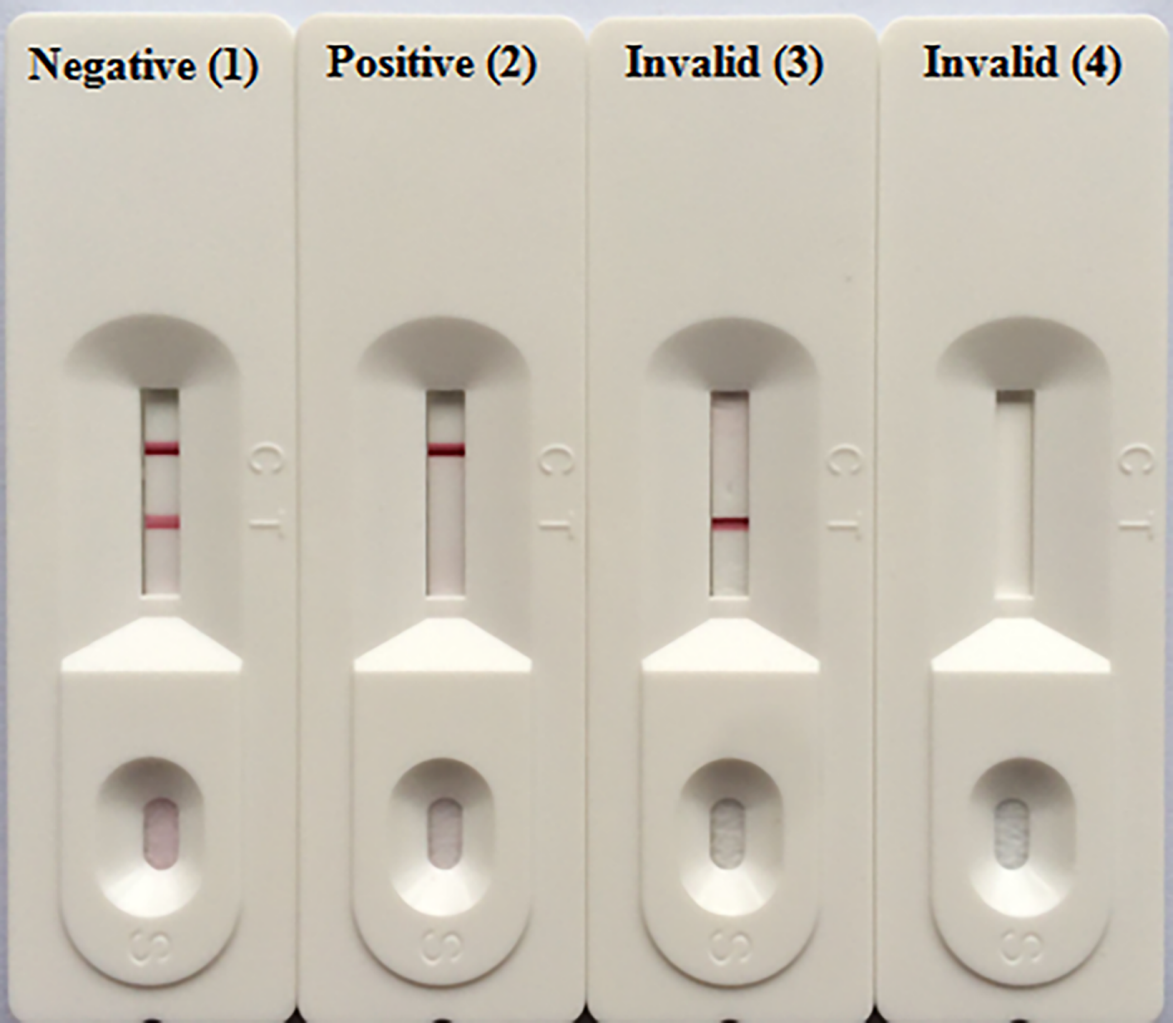
Principle of ICS test. (1). In the absence of CIT in the sample solution, red lines develop at both the control and test lines, indicating a negative readout; (2). If sufficient amount of CIT is present in the sample solution, the absence of color development on the test line indicated a positive readout; (3) & (4). No visible color development at the control line indicated invalid results.

The ICS test was based on the competitive immunoassay theory [43] and a schematic illustration was shown in Fig 2. In the absence of CIT in the sample solution, colloidal gold-labeled McAb 2B9 would migrate on the NC membrane freely, form antibody-antigen complexes with the immobilized CIT-BSA at the T line and produce a similar color intensity as that of the C line, indicating a negative result; In the presence of CIT, colloidal gold-labeled McAb 2B9 would first form antibody-antigen complexes with CIT in the sample, leaving the rest antibody to react with CIT-BSA and yield a weaker or sometimes even no T line color, this is a positive result. Color intensity on the T line is proportional to the amount of CIT in the sample. As a positive control the C line would always show a red line in any assay regardless of the CIT status, serving as a standard to ensure both strip and testing procedures are in good working conditions; If color development is absent at the C line, the tests were considered invalid.

### Specificity and sensitivity of ICS test

**Fig 3:**
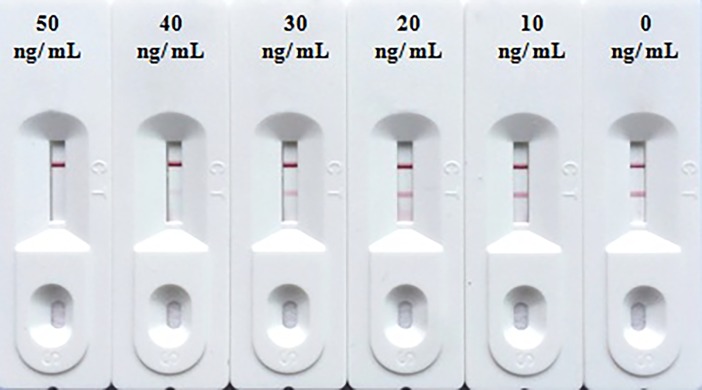
Sensitivity of the ICS. The concentrations of the toxin were 0, 10, 20, 30, 40, 50 ng/mL, respectively. Color development was inspected visually.

The detection limit of the test is defined as the lowest concentration of CIT that could result in a complete invisibility at the T line by naked eyes. Different concentrations of CIT standard solution were assayed by the ICS test to confirm the detection limit. The test results were judged by at least three individual visualizations within 5 min after the reaction. As shown in Fig 3, various concentrations of CIT standard solution (0, 10, 20, 30, 40 and 50 ng/mL) were tested by the ICS, no color development at the T line was observed at ≥ 50 ng/mL of CIT. After optimization, the detection limit of the ICS test for CIT was 50 ng/mL.

**Fig 4:**
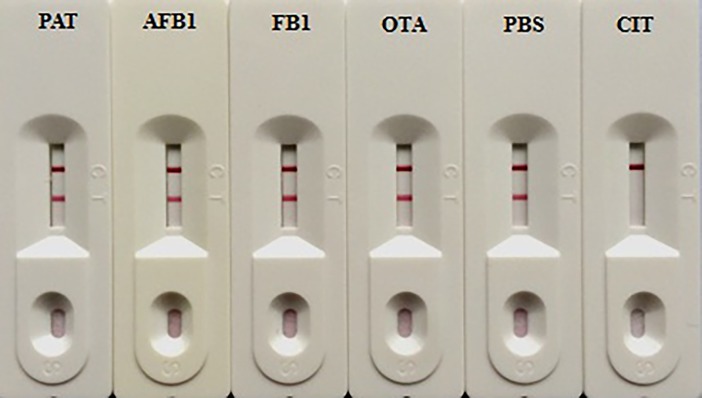
Cross-reactivity of the ICS. Patulin (PAT), Aflatoxin B1 (AFB1), Fumonisin B1 (FB1) and Ochratoxin A (OTA) were used to test the cross-reactivity. PBS and CIT were performed as negative control and positive control, respectively.

Cross reactivity of the strip was also tested with other toxins, such as Patulin (PAT), Aflatoxin B1 (AFB1), Fumonisin B1 (FB1) and Ochratoxin A (OTA). The results indicated that red color was observed in both T line and C line in samples containing other toxins, while no color development was observed in T line in sample including CIT, as shown in Fig 4. These results showed that the ICS had a specific binding ability with CIT and no cross-reaction with other related toxins, even at the concentration of 200 ng/mL.

### Samples analysis

64 natural fruit samples were collected from the different regions of Zhejiang province in 2016. ICS test, commercial ELISA kit and HPLC analysis were used to detect the spiked samples and natural samples. When the concentration of CIT was higher than 50 ng/mL, the analysis results from ICS were highly consistent with those from ELISA and HPLC (Table 1). CIT positive results included sample 2 of orange, sample 14 of ananas and sample 10 of pear which containing 85.057, 60.89 and 150.731 ng/mL, respectively, in ELISA and HPLC, and gave a positive result with only one red line on the ICS test (S2 Table). All fruit samples with CIT levels were lower than 50 ng/mL, indicating that they were negative in the ICS test. The results of the ICS test were in good agreement with those obtained from the ELISA kit and HPLC analysis. The results show that the three methods corresponded very well, and neither false-positives nor false-negatives were detected with the ICS test.

**Table 1:** Results of ICS test, ELISA and HPLC assays for CIT residues in fruit samples spiked at concentration of 30, 60, 120 ng/mL.

No. of sample	Spiked level (ng/mL)	ELISA^a^ (ng/mL) mean ± S.D.^b^	ICS test (*n* = 3)	HPLC (ng/mL) mean ± S.D.
1	30	32.203 ± 0.721	-^c^/-/-	29.902 ± 0.112
2	60	59.501 ± 0.453	+^d^/+/+	61.384 ± 0.192
3	120	122.397 ± 0.202	+/+/+	120.578 ± 0.771

^a^ Screening by Ridascreen Fast CIT ELISA kits.

^b^ Standard deviation (*n* = 3).

^c^ Negative result, T line appeared clearly.

^d^ Positive result, T line vanished.

Several classical analytical techniques for detection of CIT residues have been developed [20–23]. Although their measurements are very accurate, these methods require trained personnel, expensive instruments, tedious sample preparation, and are unsuitable for large quantity of samples on-site. The ICS test is simple, rapid and could be completed in 10 min, which has been widely used for the detection of mycotoxins on-site [26–32]. The ICS for detection of CIT residues in fruit has been developed in this study for the first time, the test could be completed in 5 min, with the detection limit of 50 ng/mL. The ICS test results were in strong agreements with the HPLC and ELISA analysis, and ICS test could be used by personnel to screen samples on-site without any training.

## Conclusion

To detect CIT residues in fruit samples, an anti-CIT McAb 2B9 with the affinity of 9.39 × 10^8^ L/moL was successful prepared and characterized, and the McAb 2B9 was applied to develop an ICS for CIT residue detection in this study. We described the development of the ICS strip in details, such as the pH of colloidal gold solution, the amount of McAb, the CIT-protein conjugates, NC membrane, conjugate pad, sample pad and extraction solution. All these materials could affect the sensitivity, signal brightness and the test time of the ICS strip. To our knowledge, this study was the first report of development of the ICS test for on-site rapid CIT detection in fruit samples. Under these optimal conditions the ICS test could be used as a qualitative and semi-quantitative tool for detection of CIT residues within 5 min with a visual detection limit of 50 ng/mL and no cross-reactivity with other mycotoxins. The results of the ICS test were in good correlation with the ELISA kit and HPLC analysis. The detection limit of the strip is content for the maximum residue limit of 50 ng/mL proposed by legislation in China. To summarize, this ICS assay could provide an alternative tool for convenient, rapid, sensitive and semi-quantitative detection of citrinin pollution residues in both laboratory and non-laboratory sites.

## Supporting information

S1 Fig Chemical structure of citrinin (CIT). (TIF)

S2 Fig The titer of the six Balb/C mice after six rounds of immunization by indirect ELISA. The serum of the non-immunized mouse was used as the negative control. All the sera were serially diluted from 1:100 to 1:204800.
(TIF)

S3 Fig Four positive hybridoma cells were screened by indirect ELISA, named 3B11, 2B9, 2C1 and 4D5. (TIF)

S4 Fig The isotypes of the four McAbs were identified by indirect ELISA. (TIF)

S5 Fig Indirect competitive ELISA analysis of the sensitivity of the four McAbs (in triplicate). Different concentrations of free CIT ranging from 1000 ng/mL to 0 were used to inhibit the binding of the McAbs to the antigen CIT-OVA. B is the OD_450nm_ at certain concentration of free CIT and B0 is the OD_450nm_ at zero concentration of free CIT.
(TIF)

S6 Fig The affinity constant *K*_*aff*_ of the McAb 2B9 was determined by indirect ELISA. The serially diluted concentrations of the antigen CIT-OVA were 1 μg/mL, 0.5 μg/mL and 0.25 μg/mL, respectively. The concentrations of the McAb 2B9 were 0.82 mg/mL, 0.41 mg/mL, 0.205 mg/mL, 0.1025 mg/mL, 0.05125 mg/mL, 0.025625 mg/mL, 0.0128125 mg/mL and 0.00640625 mg/mL, respectively.
(TIF)

S7 Fig Chromosome number of the hybridoma cell of 2B9. (TIF)

S8 Fig SDS-PAGE analysis of the purification result of McAb 2B9. Lane M: protein standards; Lane 1: purified McAb; Lane 2: unpurified ascite fluid.
(TIF)

S9 Fig The titer of the purified ascites fluids by indirect ELISA. The serum of the non-immunized mouse was used as the negative control. The purified ascites fluids were serially diluted from 1:100 to 1:6553600.
(TIF)

S1 Table Cross-reactivity of McAb 2B9 with other mycotoxins. (DOC)

S2 Table Results of ICS test, ELISA and HPLC assays for CIT residues in natural fruit samples in Zhejiang province. (DOC)

S1 File Details of McAb generation and characterization. (DOC)
