# Supplementary figures and images for: Novel monoclonal antibody-based immunochromatographic strip for detecting citrinin in fruit from Zhejiang province, China
Source: PLoS One. 2018 May 9;13(5):e0197179. doi: 10.1371/journal.pone.0197179 (PMC5942799; doi:10.1371/journal.pone.0197179)

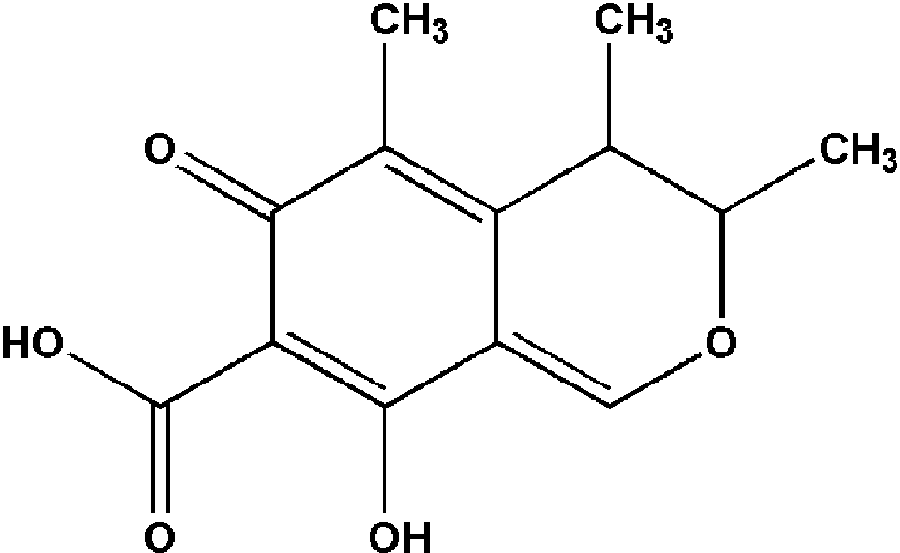

Supplement: S1 Fig — (TIF) [file pone.0197179.s001.tif]

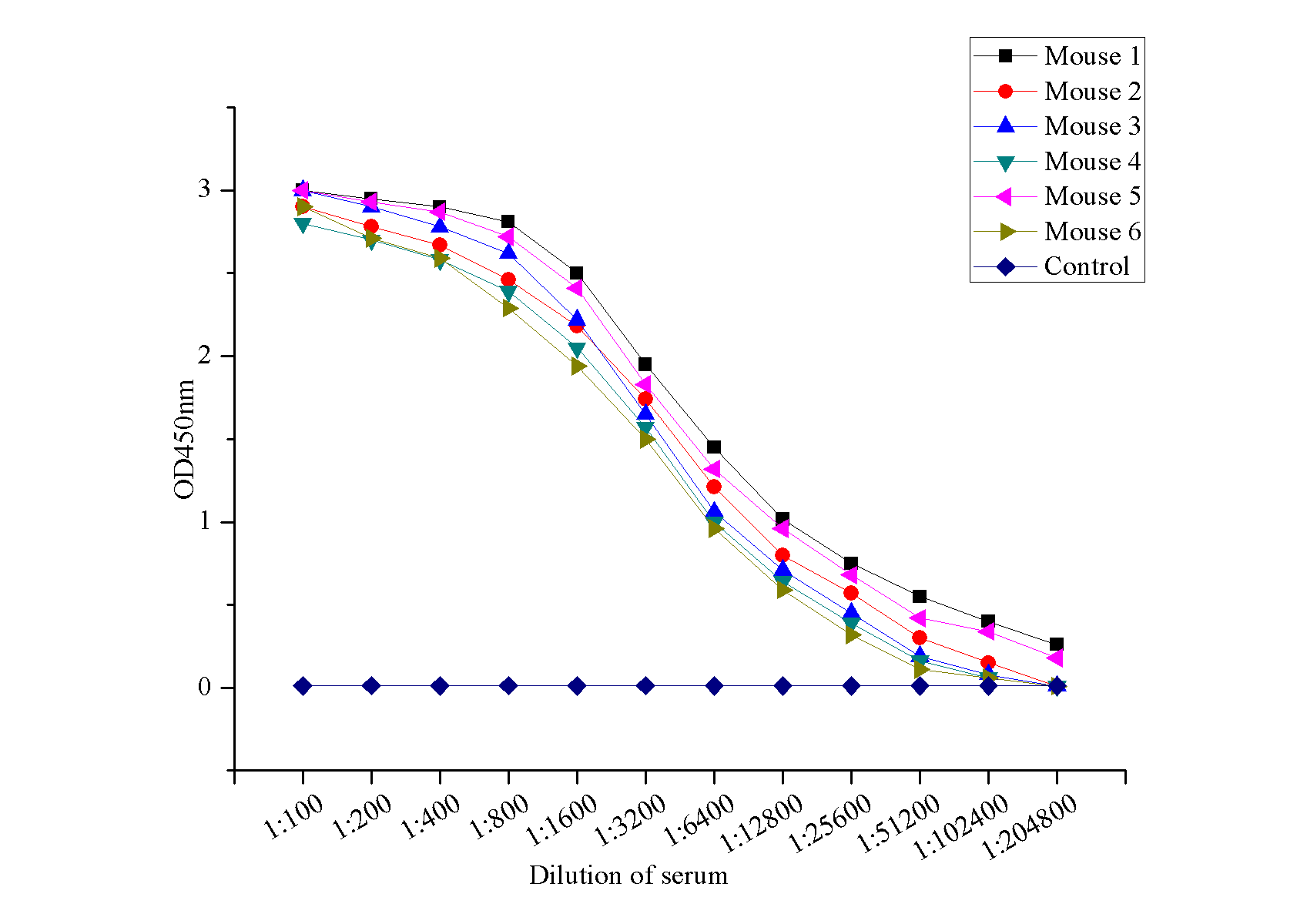

Supplement: S2 Fig — The serum of the non-immunized mouse was used as the negative control. All the sera were serially diluted from 1:100 to 1:204800. (TIF) [file pone.0197179.s002.tif]

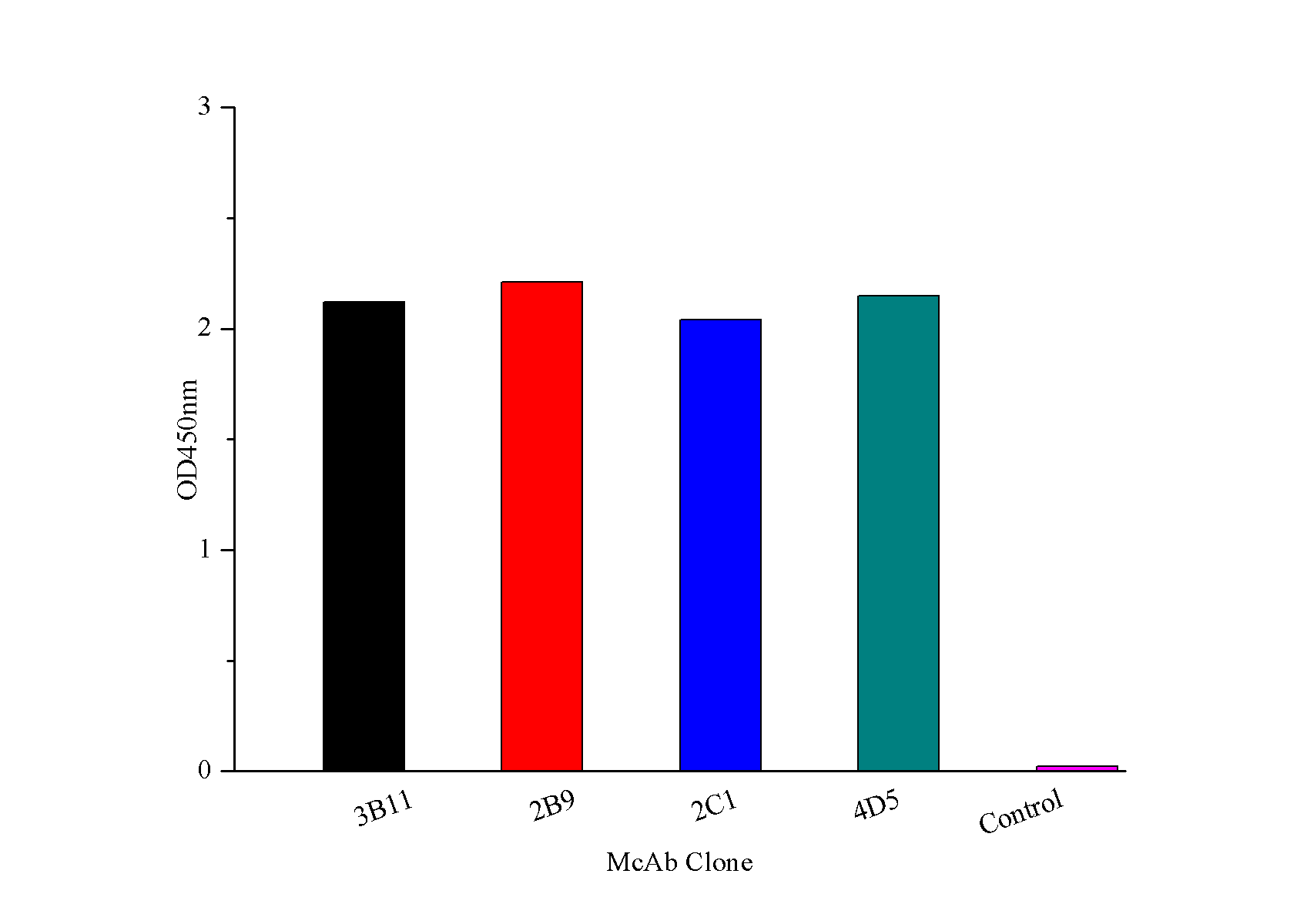

Supplement: S3 Fig — (TIF) [file pone.0197179.s003.tif]

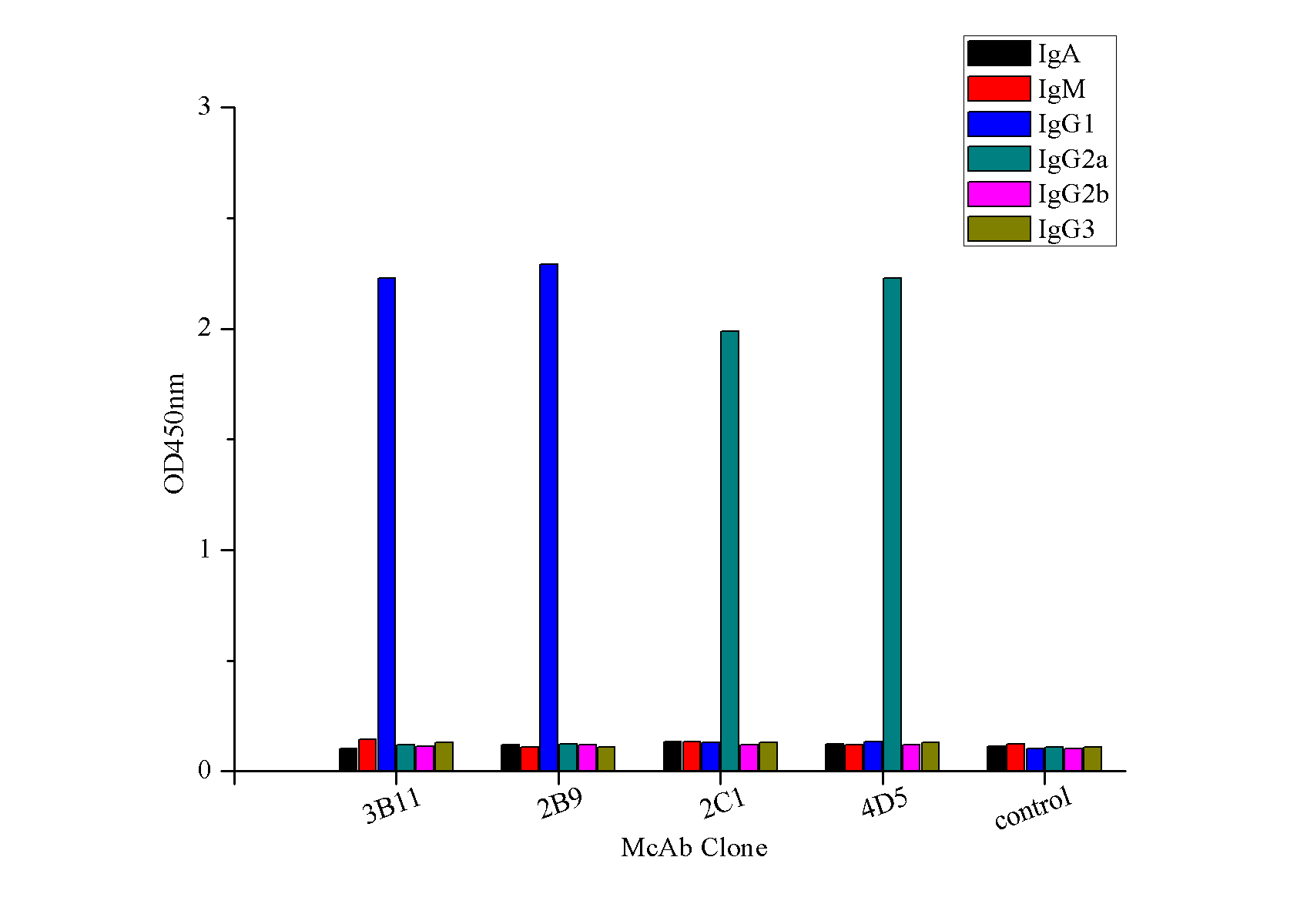

Supplement: S4 Fig — (TIF) [file pone.0197179.s004.tif]

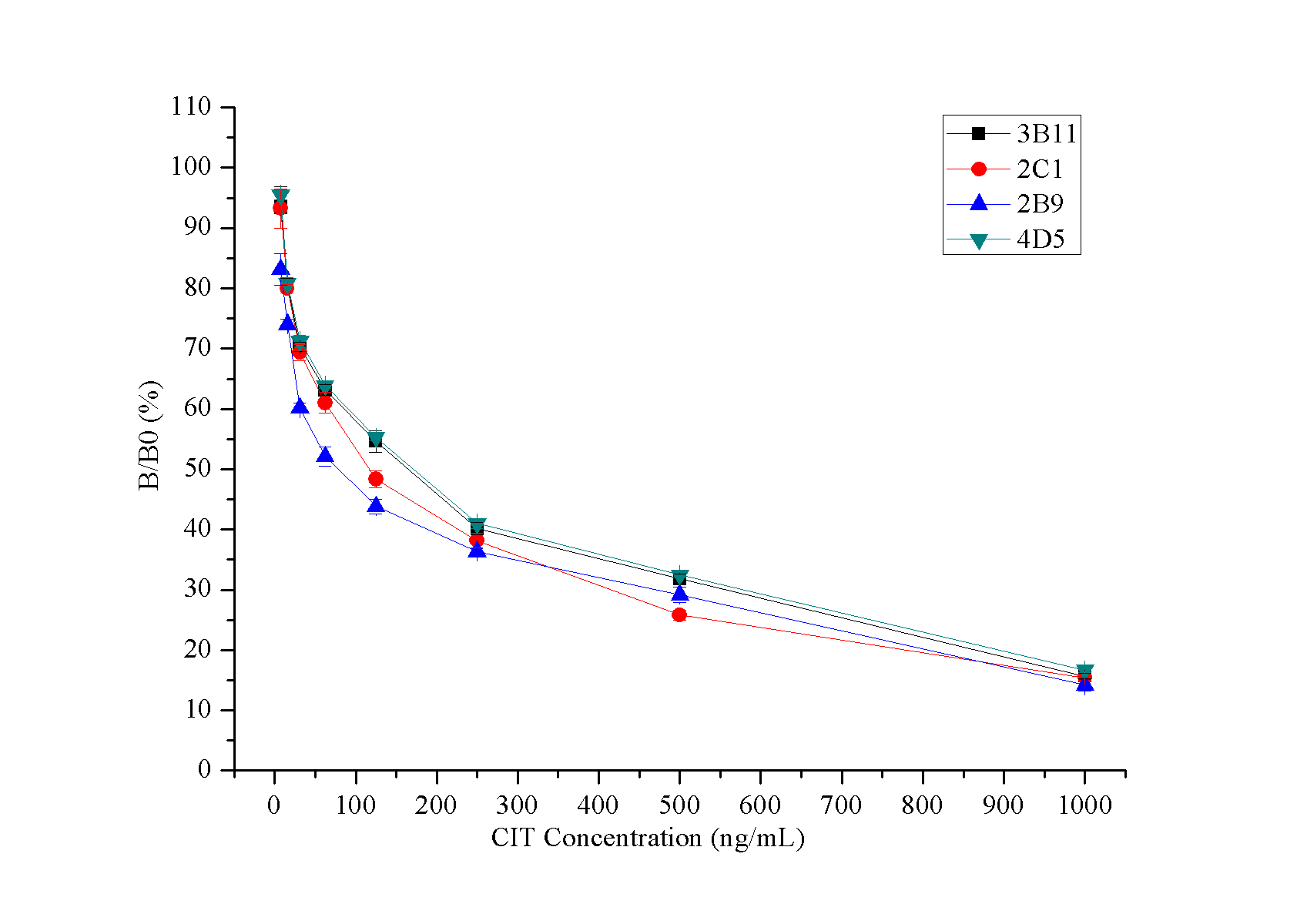

Supplement: S5 Fig — Different concentrations of free CIT ranging from 1000 ng/mL to 0 were used to inhibit the binding of the McAbs to the antigen CIT-OVA. B is the OD450nm at certain concentration of free CIT and B0 is the OD450nm at zero concentration of free CIT. (TIF) [file pone.0197179.s005.tif]

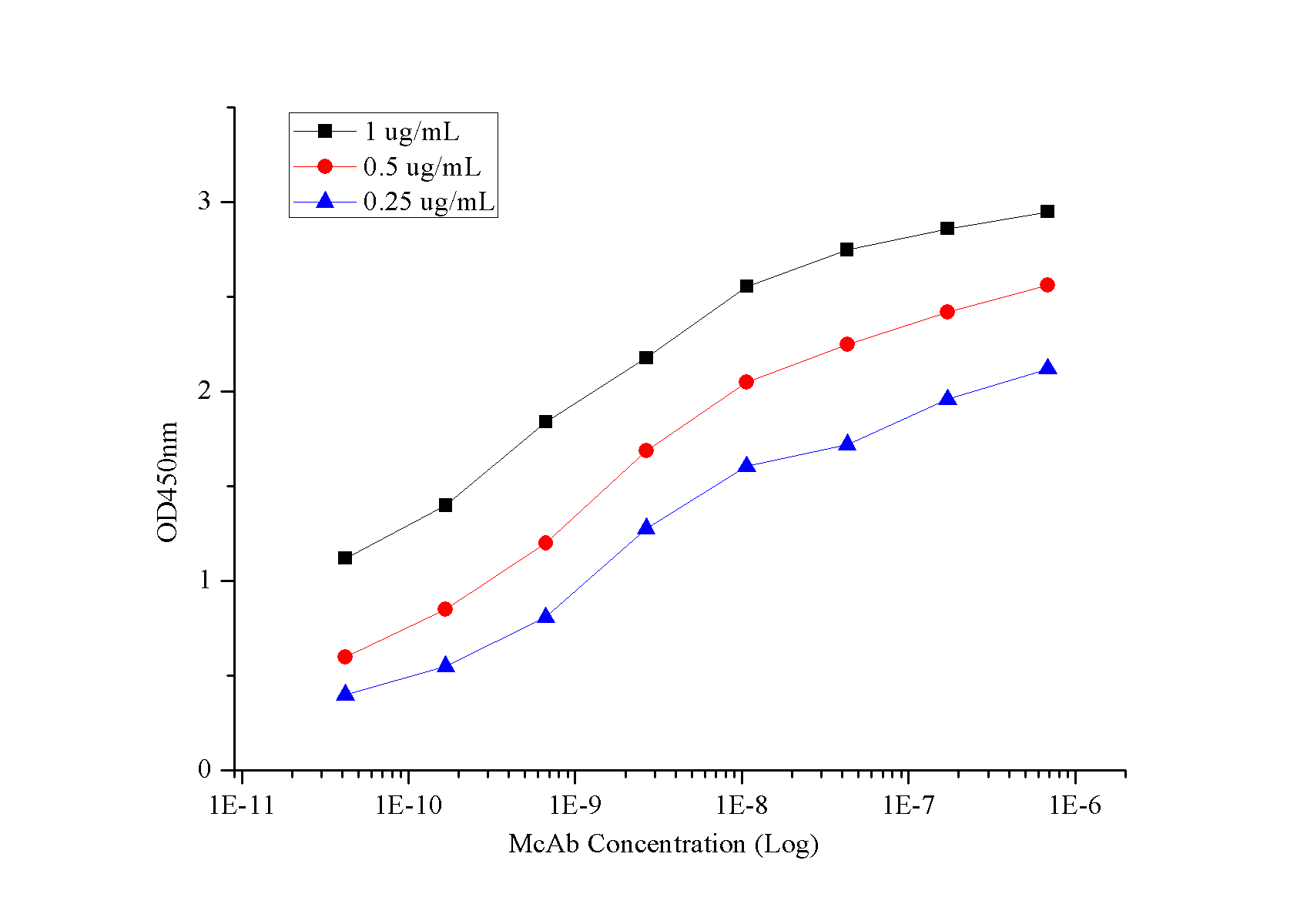

Supplement: S6 Fig — The serially diluted concentrations of the antigen CIT-OVA were 1 μg/mL, 0.5 μg/mL and 0.25 μg/mL, respectively. The concentrations of the McAb 2B9 were 0.82 mg/mL, 0.41 mg/mL, 0.205 mg/mL, 0.1025 mg/mL, 0.05125 mg/mL, 0.025625 mg/mL, 0.0128125 mg/mL and 0.00640625 mg/mL, respectively. (TIF) [file pone.0197179.s006.tif]

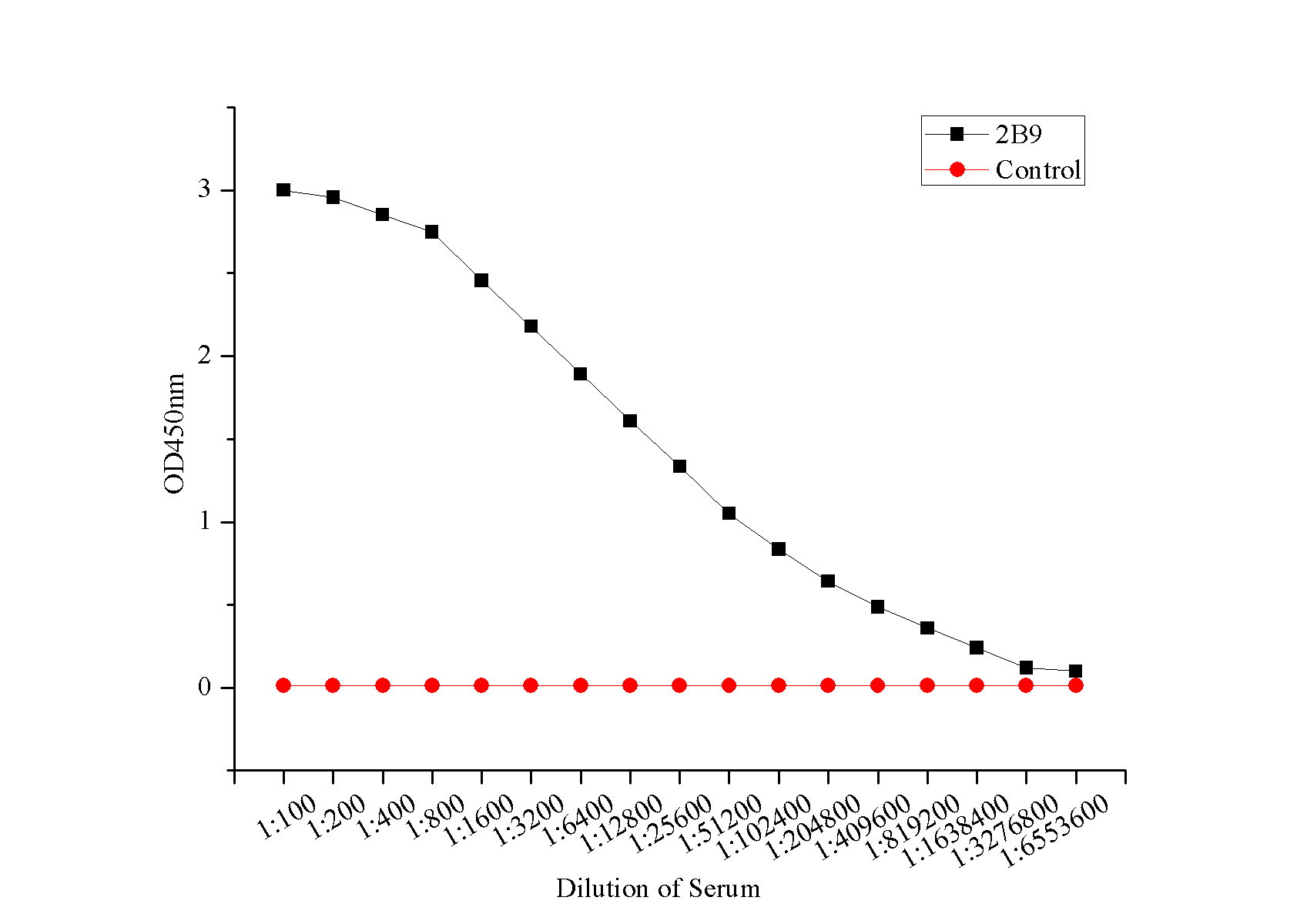

Supplement: S9 Fig — The serum of the non-immunized mouse was used as the negative control. The purified ascites fluids were serially diluted from 1:100 to 1:6553600. (TIF) [file pone.0197179.s009.tif]
