# Supplementary material for: Novel monoclonal antibody-based immunochromatographic strip for detecting citrinin in fruit from Zhejiang province, China
Source: PLoS One. 2018 May 9;13(5):e0197179. doi: 10.1371/journal.pone.0197179 (PMC5942799; doi:10.1371/journal.pone.0197179)
Supplement: S1 Table — (DOC) [file pone.0197179.s010.doc]

**S1 Table.** **Cross-reactivity of McAb 2B9 with other mycotoxins.**

| Mycotoxins | PAT | AFB1 | FB1 | OTA | CIT |
| --- | --- | --- | --- | --- | --- |
| Cross-reactivity (%) ^a^ | <0.01 | <0.01 | <0.01 | <0.01 | 100 |

The reactivity to other mycotoxins was determined by indirect competitive ELISA and the values were calculated according to the four-parameter logistic equation.

^a^ Cross-reactivity (%) = (IC_50_ of CIT/IC_50_ of other mycotoxins) × 100.
