# Supplementary material for: Novel monoclonal antibody-based immunochromatographic strip for detecting citrinin in fruit from Zhejiang province, China
Source: PLoS One. 2018 May 9;13(5):e0197179. doi: 10.1371/journal.pone.0197179 (PMC5942799; doi:10.1371/journal.pone.0197179)
Supplement: S2 Table — (DOC) [file pone.0197179.s011.doc]

**S2 T**able. Results of ICS test, ELISA and HPLC assays for CIT residues in natural fruit samples in Zhejiang province.

| **Sample NO.** | | **CIT kit^a^  (ng/mL)**  **mean ±S.D.^b^** | **HPLC (****ng/mL)**  **mean ±S.D.** | **ICS test (*n*=3)** | **Sample NO.** | | **CIT kit^a^  (ng/mL)**  **mean ±S.D.** | **HPLC (ng/mL)**  **mean ±S.D.** | **ICS test (*n*=3)** |
| --- | --- | --- | --- | --- | --- | --- | --- | --- | --- |
| 1 | Orange | < LOD^c^ | ND^d^ | -e/-/- | 1 | Apple | < LOD | ND | -/-/- |
| 2 | Orange | 88.351 ± 0.235 | 85.057 ± 0.191 | +/+/+ | 2 | Apple | < LOD | ND | -/-/- |
| 3 | Orange | < LOD | ND | -/-/- | 3 | Apple | < LOD | 1.792 ± 0.309 | -/-/- |
| 4 | Orange | < LOD | 5.101 ± 0.211 | -/-/- | 4 | Apple | < LOD | ND | -/-/- |
| 5 | Orange | < LOD | ND | -/-/- | 5 | Apple | < LOD | ND | -/-/- |
| 6 | Orange | < LOD | ND | -/-/- | 6 | Apple | < LOD | ND | -/-/- |
| 7 | Orange | < LOD | ND | -/-/- | 7 | Apple | < LOD | ND | -/-/- |
| 8 | Orange | 19.536 ± 0.831 | 17.364 ± 0.374 | -/-/- | 8 | Apple | < LOD | ND | -/-/- |
| 9 | Orange | < LOD | ND | -/-/- | 9 | Apple | < LOD | ND | -/-/- |
| 10 | Orange | < LOD | ND | -/-/- | 10 | Apple | < LOD | ND | -/-/- |
| 11 | Orange | < LOD | ND | -/-/- | 11 | Apple | 21.781 ± 0.908 | 18.231 ± 0.298 | -/-/- |
| 12 | Orange | < LOD | 9.873 ± 0.293 | -/-/- | 12 | Apple | < LOD | ND | -/-/- |
| 13 | Orange | < LOD | ND | -/-/- | 13 | Apple | < LOD | ND | -/-/- |
| 14 | Orange | < LOD | ND | -/-/- | 14 | Apple | < LOD | ND | -/-/- |
| 15 | Orange | < LOD | ND | -/-/- | 15 | Apple | < LOD | ND | -/-/- |
| 16 | Orange | < LOD | ND | -/-/- | 16 | Apple | < LOD | ND | -/-/- |
| 1 | Ananas | < LOD | ND | -/-/- | 1 | Pear | < LOD | ND | -/-/- |
| 2 | Ananas | < LOD | ND | -/-/- | 2 | Pear | < LOD | ND | -/-/- |
| 3 | Ananas | < LOD | 1.991 ± 0.287 | -/-/- | 3 | Pear | < LOD | 2.892 ± 0.773 | -/-/- |
| 4 | Ananas | < LOD | ND | -/-/- | 4 | Pear | < LOD | ND | -/-/- |
| 5 | Ananas | < LOD | ND | -/-/- | 5 | Pear | < LOD | ND | -/-/- |
| 6 | Ananas | < LOD | ND | -/-/- | 6 | Pear | < LOD | ND | -/-/- |
| 7 | Ananas | < LOD | ND | -/-/- | 7 | Pear | < LOD | ND | -/-/- |
| 8 | Ananas | < LOD | 7.911 ± 0.891 | -/-/- | 8 | Pear | < LOD | ND | -/-/- |
| 9 | Ananas | < LOD | ND | -/-/- | 9 | Pear | < LOD | ND | -/-/- |
| 10 | Ananas | < LOD | ND | -/-/- | 10 | Pear | 145.983 ± 0.184 | 150.731 ± 0.351 | +/+/+ |
| 11 | Ananas | < LOD | ND | -/-/- | 11 | Pear | < LOD | ND | -/-/- |
| 12 | Ananas | < LOD | ND | -/-/- | 12 | Pear | < LOD | ND | -/-/- |
| 13 | Ananas | < LOD | ND | -/-/- | 13 | Pear | < LOD | 4.478 ± 0.092 | -/-/- |
| 14 | Ananas | 65.843 ± 0.132 | 60.890 ± 0.261 | +f/+/+ | 14 | Pear | < LOD | ND | -/-/- |
| 15 | Ananas | < LOD | ND | -/-/- | 15 | Pear | < LOD | ND | -/-/- |
| 16 | Ananas | < LOD | ND | -/-/- | 16 | Pear | < LOD | ND | -/-/- |

^a^ Screening by Ridascreen Fast CIT ELISA kits.

^b^ Standard deviation (*n* = 3).

^c^ Limit of detection of CIT, 15 ng/mL.

^d^ Not detected (ND).

^e^ Negative result, T line appeared clearly.

^f^ Positive result, T line disappeared.
