# Supplementary material for: Novel monoclonal antibody-based immunochromatographic strip for detecting citrinin in fruit from Zhejiang province, China
Source: PLoS One. 2018 May 9;13(5):e0197179. doi: 10.1371/journal.pone.0197179 (PMC5942799; doi:10.1371/journal.pone.0197179)
Supplement: S1 File — (DOC) [file pone.0197179.s012.doc]

**Supporting information S1: Details of McAb generation and characterization**

**Novel monoclonal antibody-based immunochromatographic strip for detecting citrinin in fruit from Zhejiang province, China**

Haiwei Cheng^1,2,3^, Yi Yang^1^, Yifei Chen^1^, Xueqiu Chen^1^, Zizheng Cai^4^, Aifang Du^1^,*

^1^ Institute of Preventive Veterinary Medicine & Zhejiang Provincial Key Laboratory of Preventive Veterinary Medicine, College of Animal Sciences, Zhejiang University, Hangzhou 310058, China.

^2^ National Research Center of Engineering and Technology for Veterinary Biologicals, Ministry of Agriculture, Key Laboratory of Veterinary Biological Engineering and Technology, Jiangsu Academy of Agricultural Sciences, Nanjing 210014, China

^3^ Jiangsu Co-innovation Center for Prevention and Control of Important Animal Infectious Diseases and Zoonoses, Yangzhou 225009, China

^4^ Nanjing Agricultural University, Nanjing 210095, China

* Corresponding author

*E-mail address*: [afdu@zju.edu.cn](mailto:afdu@zju.edu.cn) (AD)

**Methods**

**Preparation of antigens**

CIT is non-immunogenic as the character of hapten, and the CIT-BSA and CIT-OVA conjugates were prepared according to the method described in Cheng et al. (2015) to overcome the limit in the immune response of CIT.

**Immunization of mice**

Six female Balb/C mice (6-8 weeks old) were injected multi-point subcutaneously with 50 μg CIT-BSA emulsified with the equal amount of Freund’s complete adjuvant, respectively. Then the mice were immunized with 50 μg CIT-BSA emulsified with the same volume of Freund’s incomplete adjuvant at the interval of 2 weeks. Sera of the immunized mice were collected 7 days after each immunization and monitored for anti-CIT activity with CIT-OVA by ELISA (Li et al., 2011). After 6 times injections, the most responsive mouse was chosen and received a final intraperitoneally booster of 50 μg CIT-BSA 3 days before cell fusion.

**Determination of antibody titers**

Antibody titer was determined by indirect ELISA. Briefly, microtiter plates were coated with 5 μg/mL CIT-OVA in 0.05 M carbonate/bicarbonate buffer (pH 9.6) at 4 ^o^C overnight. The microtiter plates were washed with PBST (PBS containing 0.05% Tween 20(v/v), pH 7.4) for five times and blocked with 200 μL of 5% skimmed milk at 37 ^o^C for 1 h. After five washings, the serially diluted antibody was added and incubated at 37 ^o^C for 1 h, while the serum antibody of the non-immunized mouse was used as the negative control. After five washings, HRP-labeled goat anti-mouse IgG (H+L) was added and incubated at 37 ^o^C for 1 h. TMB was used as the substrate solution and incubated at 37 ^o^C for 15 min after five washings. The reaction was stopped with 2 M H_2_SO_4_ and the absorbance was read at 450 nm.

**Cell fusion and hybridoma screening**

Anti-CIT McAb was prepared according to the method described by Kohler and Milstein (1975) with small modifications. The splenocytes isolated from the immunized mouse were fused with the SP2/0 myeloma cells at a ratio of 5: 1 by polyethylene glycol (PEG) 1450 (Sigma, USA) (Kohler and Milstein, 1975). After fusion, the cells were cultured in the 96-well microtiter plates with HAT medium at 37 ^o^C under an atmosphere of 5% CO_2_ and 95% humidity. Ten days later, the supernatant of each well was assessed to screen antibody against CIT using the antigen CIT-OVA by indirect ELISA as described above. The positive hybridoma cells were subcloned by limiting dilution (Davis et al., 1982).

**Characterization of McAb**

The isotypes of the McAbs were identified according to the instructions of the McAb isotyping kit and the assay was performed with the antibody isotyping reagents by indirect ELISA.

Indirect competitive ELISA was performed to determine the sensitivity and specificity of the McAbs (Rath et al., 1988). Different concentrations of free CIT were prepared with PBST containing 10% methanol (1000 ng/mL, 500 ng/mL, 250 ng/mL, 125 ng/mL, 62.5 ng/mL, 31.25 ng/mL, 15.625 ng/mL, 7.8125 ng/mL and 0 ng/mL) and the McAbs were preincubated with different concentrations of free CIT, respectively. The mix was added to microtiter wells coated with 5 μg/mL CIT-OVA and incubated for 1 h at 37 ^o^C. Then the assay was performed as the indirect ELISA described above. The binding rate (B/B0) was calculated according to the following equation: B/B0 (%) = (OD_450nm_ at certain concentration of free CIT/OD_450nm_ at zero concentration of free CIT) × 100.

Patulin (PAT), Aflatoxin B1 (AFB1), Fumonisin B1 (FB1) and Ochratoxin A (OTA) (Sigma, USA) were used to evaluate the cross-reactivity (CR) of the McAb and the CR values were calculated according to the following equation: CR (%) = (IC_50_ of CIT/IC_50_ of other mycotoxins) × 100. IC_50_ was the concentration of free mycotoxin at which binding of the McAb to CIT-OVA was inhibited by 50%.

The affinity constant *K_aff_* of the McAb was determined by indirect ELISA according to the method of Beatty et al. (1987), which both the antigen CIT-OVA and the McAb were serially diluted. The K*_aff_* would be calculated as the following equation: *K_aff_*=(n-1)/2(n [Ab]^1^_t_-[Ab]^2^_t_). [Ab]_t_ was the concentration of McAb at 50% of the max OD_450nm_. n = [Ag]^1^_t_ / [Ag]^2^_t_, [Ag]_t_ was the concentration of coating antigen.

Chromosomes of the hybridoma cells were analyzed according to the method of Ian (1983) with little modifications. About 1×10^6^ hybridoma cells were cultured with 0.04 μg/mL colchicine for 5~7 hours. KCl (10 mL, 0.075 M) was added and incubated at 37 ^o^C for 45 min. The cells were fixed with the freshly prepared fixative (methanol to glacial acetic acid with a ratio of 3:1) at 4 ^o^C overnight. The chromosomes were observed by microscope after stained with Giemsa dye liquor.

Ascites fluids were produced in the paraffin-primed Balb/C mice and the anti-CIT McAb was purified by the Protein-G Sepharose Fast Flow Column according to the instructions of the manufacturer. SDS-PAGE was performed to determine the purification status of ascites fluid and indirect ELISA was used to assess the titer of purified ascites fluid according to the method described above.

**References**

Beatty, J.D., Beatty, B.G., & Vlahos, W.G. (1987). Measurement of monoclonal antibody affinity by non-competitive enzyme immunoassay. *Journal of Immunological Methods*, *100*, 173-179.

Cheng, H. W., Chen, Y. F., Yang, Y., Chen, X. Q., Guo, X. L., & Du, A.F. (2015). Characterization of anti-Citrinin specific scFvs selected from a non-immunized mouse splenocytes by eukaryotic Ribosome Display. *PLoS One*, *10* (7), e0131482.

Davis, J.M., Pennington, J.E., Kubler, A.M., & Conscience, J.F. (1982). A simple, single-step technique for selecting and cloning hybridomas for the production of monoclonal antibodies. *Journal of Immunological Methods*, *50*, 161–171.

Ian, F. R. (1983). Culture of Animal Cells: A Manual of Basic Technique and Sp. Alan R. Liss.

Köhler, G., & Milstein, C. (1975). Continuous cultures of fused cells secreting antibody of predefined specificity. *Nature, 256*, 495−497.

Li, Y. N., Wang, Y. Y., & Guo, Y. H. (2011). An indirect competitive ELISA for determination of citrinin. *Journal of Food Safety, 31*, 497-504.

Rath, S., Stanley, C. M., & Steward, M. W. (1988). An inhibition enzyme immunoassay for estimating relative antibody affinity and affinity heterogeneity. *Journal of Immunological Methods*, *106*, 245–249.
